# A Review on Major Pathways Leading to Peritoneal Fibrosis in Patients Receiving Continuous Peritoneal Dialysis

**DOI:** 10.7759/cureus.31799

**Published:** 2022-11-22

**Authors:** Sogand Taheri, Suvedha S Thiagaraj, Twisha S Shukla, Sai Dheeraj Gutlapalli, Hadi Farhat, Kanmani Muthiah, Namratha Pallipamu, Pousette Hamid

**Affiliations:** 1 Nephrology, California Institute of Behavioral Neurosciences and Psychology, Fairfield, USA; 2 Internal Medicine, California Institute of Behavioral Neurosciences and Psychology, Fairfield, USA; 3 Pediatric, California Institute of Behavioral Neurosciences and Psychology, Fairfield, USA; 4 Cardiology and Rheumatology, California Institute of Behavioral Neurosciences and Psychology, Fairfield, USA; 5 Cardiology and Rheumatology, University of Balamand, Beirut, LBN; 6 Neurology, California Institute of Behavioral Neurosciences and Psychology, Fairfield, USA

**Keywords:** peritoneal fibrosis, mesothelial to mesenchymal transitioning, mmt, tgf-β1, transforming growth factor-beta (tgf-β) receptor type 1, continuous ambulatory peritoneal dialysis, peritoneal dialysis complication, encapsulating peritoneal sclerosis, peritoneal dialysis (pd)

## Abstract

Peritoneal fibrosis (PF) is the most important complication of peritoneal dialysis (PD) that may arise among patients receiving continuous ambulatory peritoneal dialysis (CAPD). PF is a complex process, and many factors contribute to the formation of fibrosis. PD solutions with high glucose content, chronic inflammation, inflammatory cytokines, angiogenesis, and mesothelial to mesenchymal transition (MMT) are factors contributing to the fibrosis of the peritoneum. These factors, as well as stress-induced fibrosis, are going to be discussed further in this article.

Although most experimental models are promising in preventing or delaying PD-related fibrosis, most of these recommended treatment options require further research. The lack of sufficient data from real PD patients and many inconclusive data make clinicians depend on conservative treatment. New therapeutics are indeed required for the management of patients undergoing PD to prevent the dreaded complication that may arise from continuous PD. Newer PD solutions are needed to improve survival and minimize the complication associated with PD. Recently, newer PD solutions have been shown to improve patient survival and peritoneal viability and reduce this complication that may arise as a result of continuous PD.

## Introduction and background

With over two million individuals suffering from end-stage renal disease (ESRD) worldwide [1], it is worth elaborating on peritoneal dialysis (PD) as one of the two methods of dialysis used by ESRD patients. Although PD is less physiologically stressful [2] and economically more feasible [3] than hemodialysis (HD), the prevalence of PD among maintenance dialysis patients in the United States is much lower than in the rest of the world. Per available data, PD prevalence was 6.9% and 10.1% in 2009 and 2017, respectively, among maintenance dialysis patients in United States [4].

One advantage of PD is that it can be done at home, giving individuals greater flexibility. Individuals no longer need to go to the dialysis center. Given the current social dynamics of the post-coronavirus disease 2019 (COVID-19) pandemic, PD may be a preferred mode of dialysis for many ESRD individuals as it can be done in the comfort of their homes.

To prepare the patients though, intraperitoneal access, a catheter, is placed [2]. PD solutions are then used to run their course through this access. PD solutions have three major components: an osmotic agent, electrolytes, and a buffer [5]. In order to properly remove excess fluid, an osmotic agent such as glucose is used to force the excess fluid out. Other components of PD solutions are electrolytes at a concentration equal to the physiological level and a buffer such as bicarbonate and/or lactate [6].

Despite being an economically feasible and accessible mode of renal replacement therapy, PD is not without complications. The most important and common complication of PD is peritonitis and infection on the site of catheter insertion. Another complication of PD is peritoneal fibrosis (PF), which can arise as a result of continuous ambulatory peritoneal dialysis (CAPD).

Encapsulating peritoneal sclerosis (EPS) is another reported complication of long-term PD. Although rare, EPS happens as a result of the thickening and fibrosis of the peritoneal membrane resulting in the obstructions of part or all of the small bowel [7]. Similar to PF, EPS may arise from the fibrosis of the peritoneal membrane among patients with CAPD. Same factors such as exposure to high-glucose-containing PD solutions, inflammatory cytokines, and repeated bouts of peritonitis are among the top leading causes of EPS [8].

PF is a complex process, and many factors may contribute to the formation of fibrosis. Studies over the last two decades have reported that the following factors contribute to the fibrosis of the peritoneal membrane. PD solutions with high glucose content, inflammation, inflammatory cytokines, angiogenesis, and mesothelial to mesenchymal transition (MMT) are factors contributing to the fibrosis of the peritoneal lining. These factors are going to be discussed further in this article. Also, through this article, we are reviewing what the future may bring in terms of the management of patients with PD.

## Review

PD depends on the permeability of the peritoneal lining to be effective. Therefore, changes in peritoneal morphology that may arise from an uninterrupted and continuous PD can be detrimental. These morphological changes were observed on biopsies from individuals going through continuous PD [9]. Structural and functional deterioration of the peritoneal membrane is associated with proliferative processes of the peritoneum leading to fibrosis among individuals with continuous PD [9]. Therefore, the viability of the peritoneal membrane is crucial [10] for the proper removal of solutes and excess fluid and retaining its function.

It is important to note that the terms peritoneal sclerosis, peritoneal fibrosis, and EPS have been used interchangeably by various articles, and there are debates on whether these are part of one disease process that has exhibited differently, or they are different disease processes although they all have the same underlying pathology [11] that led to fibrosis.

The term fibrosis refers to the increase in the thickness of the peritoneum. According to Williams et al. [12], peritoneal biopsies obtained from individuals going through PD were compared to a group receiving HD, as well as a group of healthy individuals. Then, the thickness of the sub-mesothelial layer was measured in micrometer (μm). This study provided evidence that the median thickness of the sub-mesothelial layer was 270 μm for patients undergoing continuous PD compared to normal individuals and patients undergoing hemodialysis, which were 50 μm and 150 μm, respectively [12]. The thickness observed among individuals going through PD is referred to as fibrosis, which is often associated with continuous PD.

Peritoneal fibrosis is a complex process, which comprises many factors. Studies over the last two decades have reported that the following factors contribute to the fibrosis of the peritoneal membrane: PD solutions with high glucose content, MMT, inflammation, inflammatory cytokines, and angiogenesis. Each of these factors has its cascade of events, and stress-induced fibrosis is going to be discussed further in the following sections.

Stress-induced fibrosis

Initially, observed changes in the peritoneal membrane were determined to be related to volume stress-induced endothelin-1 (ET-1) release. ET-1 release contributes to structural alteration of the peritoneal membrane in long-term peritoneal dialysis leading to the alteration of the peritoneal membrane in long-term peritoneal dialysis [9]. Considering this fact, the use of bosentan or other (ET-1) inhibitors may be a useful therapeutic to prevent further fibrosis of the peritoneal membrane. The administration of bosentan, ET receptor antagonist, decreases PD-induced peritoneal fibrosis [13] and helped retain peritoneal function.

Fibrosis as a result of PD solutions with high glucose content

Prolonged exposure to PD solutions, especially the ones with high glucose content, is shown to be correlated with peritoneal fibrosis [14]. High glucose content induces mesothelial cells to produce transforming growth factor-beta (TGF-β) [15]. An analysis of 19,828 diabetic patients with PD has demonstrated that exposure to glucose upregulated dipeptidyl peptidase 4 (DPP4) [16]. The upregulation of dipeptidyl peptidase 4 (DPP4) promoted mesothelial to mesenchymal transition (MMT), which refers to the transition of the mesothelial membranes to the mesenchymal cells. DPP4 also has a role in promoting inflammation by inducing small mothers against decapentaplegic 3 (SMAD3) through TGF-β activation. A detailed discussion of TGF-β and SMAD3 activation and how it leads to fibrosis and MMT is presented in the following paragraphs. This clinical observational study of PD patients has shown that DPP4 enzyme played a major role in peritoneal fibrosis and deterioration. Therefore, the use of DPP4 inhibitors can help prevent the MMT [16] and prevent peritoneal fibrosis as DPP4 inhibition holds antifibrotic effects [17].

PD solutions have three components: electrolyte at the physiological concentration, buffer such as bicarbonate and/or lactate, and finally an osmotic agent to remove excess fluid. Newer PD solutions are suggested to improve peritoneal viability and reduce the complication that may arise as a result of continuous PD. Some of these newer PD solutions contain fewer glucose degradation products (GDP) or icodextrin [18], despite the effectiveness of icodextrin and improving patients’ survival [19]. Also, newer PD solutions contain higher molecular osmotic agents or amino acids [18].

Newer PD solutions have been shown to improve peritoneal viability and reduce the complication that may arise as a result of continuous PD. Some of these newer PD solutions contain alternative buffers, a higher pH, and fewer GDP or ones that contain higher molecular osmotic agents such as icodextrin or amino acids [18], while other studies have used neutral pH solutions.

According to a randomized controlled trial (RCT), neutral pH and low GDP PD solution have improved the residual renal function (RRF) [20]. Although the effects of either neutral pH, low GDP solution, or icodextrin on patient survival remain inconclusive, further research is required to eliminate any uncertainty. More improved PD solutions are yet to be available. To improve patients’ survival and optimize the clinical outcome, newer PD solutions are suggested. Among different solutions, PD solutions containing different osmotic agents such as xylitol and L-carnitine have been experimented. PD solution containing L-carnitine has demonstrated better preservation of urine volume and maintenance of residual kidney function [21].

Mesothelial to mesenchymal transition (MMT)

**Figure 1:**
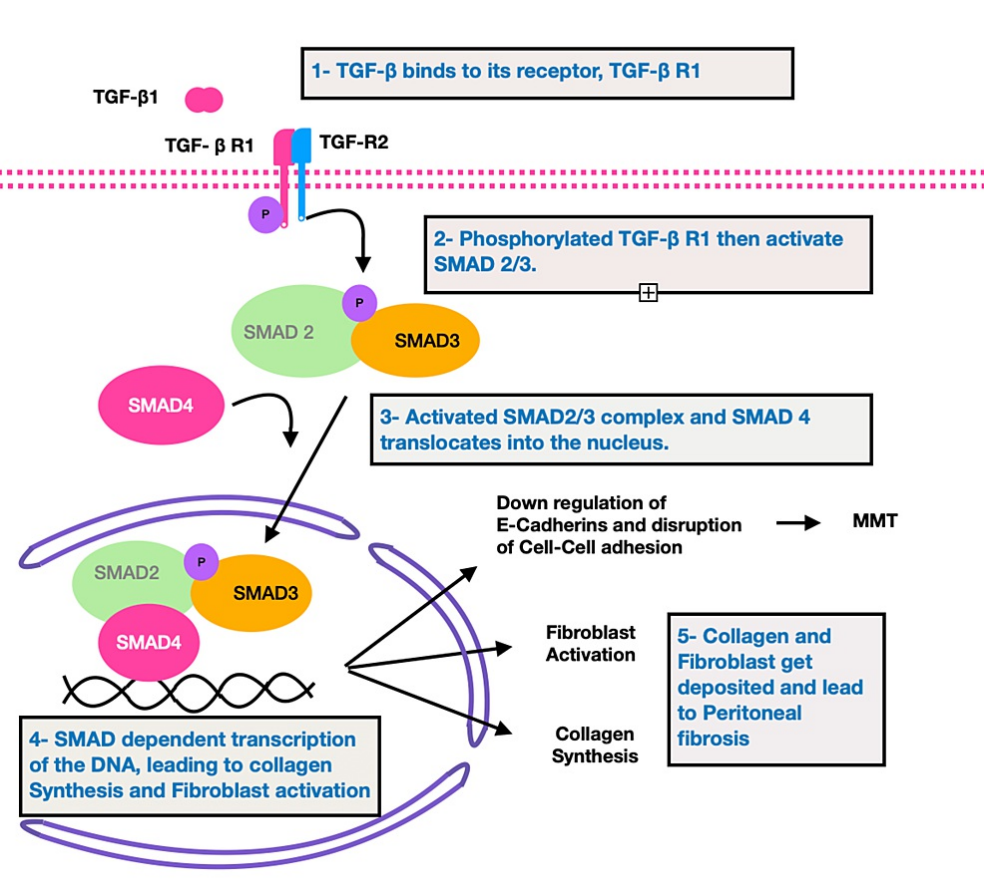
TGF-β-Induced Peritoneal Fibrosis As Illustrated above, peritoneal fibrosis is induced by the activation of TGF-β and the translocation of SMAD complex into the nucleus. These steps are the following: (1) TGF-β binds to its receptor, TGF-β R1; (2) phosphorylated TGF-β R1 then activates SMAD2/3; (3) activated SMAD2/3 complex together with SMAD4 translocates into the nucleus and leads to SMAD-dependent transcription of the DNA; (4) SMAD-dependent transcription of the DNA leads to collagen synthesis and fibroblast activation; and (5) collagen and fibroblast deposition lead to peritoneal fibrosis TGF-β: transforming growth factor-beta; TGF-β R1/2: transforming growth factor-beta receptor 1/2; SMAD: small mothers against decapentaplegic; MMT: mesothelial to mesenchymal transition

The mesothelial membrane of the peritoneum consists of a parietal and visceral layer covering the body wall and the internal organs, respectively. Morphological changes in the peritoneum are the thickening of the subendothelial layer [12] and the presence of collagen type I and fibronectin as shown on the histological samples obtained from the peritoneal cavity of patients with fibrosis [22]. MMT is characterized by the loss of cell polarity and the disruption of cell to cell adhesion leading to cell migration [23]. Other findings include the loss of the expression of E-cadherins, indicating the loss of epithelial characteristics. At the same time, the expression of the mesenchymal markers such as N-cadherin, vimentin, and α-smooth muscle actin (α-SMA) [24] is indicative of MMT.

transforming growth factor-β 1 (TGF-β1) is a principal mediator of MMT. Once released, TGF-β1 binds to its receptor, TGF-β receptor 1 (TGF-β R1). Phosphorylated TGF-β R1 then activates SMAD2/3. SMAD2/3 complex then gets translocated into the nucleus and leads to SMAD-dependent transcription of the DNA [25] leading to collagen synthesis and fibroblast activation. Collagen and fibroblasts get deposited and lead to PF, as illustrated in Figure 1.

TGF-β1 regulates a wide range of biological responses. Once activated, TGF-β1 can lead to the disruption of cell junctions and the downregulation of calcium-dependent adhesion molecules (E-cadherins). The activation of TGF-β1 can also cause the loss of cell polarity. Together, these two events, the loss of cell polarity and cell junction disruption, can lead to cell migration [25]. TGF-β1 inhibition was postulated to cause the progression of peritoneal sclerosis and peritoneal fibrosis. This opens doors to the use of agents that inhibit TGF-β1 binding to its receptor or a decoy to prevent proliferation and MMT.

Among patients receiving PD, the inhibition of TGF-β1 activation and disrupting the SMAD signaling pathway have shown promising results in animal models. Further research is still required to show the efficacy of these agents on patients receiving PD. According to an experiment performed on human peritoneal mesothelial cells (HPMCs), TGF-β1 inhibition has shown the attenuation of peritoneal fibrosis and reversed TGF-β-mediated changes such as thickening and fibrosis of the peritoneum [22].

Inflammation and recurrent peritonitis

Inflammation and peritonitis are other variables contributing to the formation of fibrosis. According to a study, long-term PD may lead to fibrosis, which is mediated by transforming growth factor-β 1 (TGF-β1) and the expression of vascular endothelial growth factor A (VEGF-A). TGF-β1 induces VEGF-A expression, which can lead to lymphangiogenesis [26] and angiogenesis. The results from this finding suggest the involvement of the TGF-β1-VEGF-A pathway as a key role in fibrosis-associated peritoneal neoangiogenesis [14].

At the same time, TGF-β can mediate the repression of E-cadherin, which are calcium-dependent cell adhesion molecules (CAMs), and results in the translocation of β-catenin, a component of the CAM complex, into the cytoplasm and eventually leading to MMT [27]. E-cadherin’s downregulation is induced by TGF‐β. E-cadherin’s downregulation is thought to be involved in mesenchymal migration and MMT.

A new therapeutic such as nintedanib may be helpful in preventing the TGF-β1-induced fibrosis. Nintedanib is an approved treatment for idiopathic pulmonary fibrosis (IPF), and it is known for its antifibrotic effect [28]. Nintedanib prevents TGF-β1 activation. Hence, nintedanib prevents TGF-β1-induced downregulation of E-cadherins, reduces angiogenesis, and suppresses the production of cytokines in the peritoneum [29].

Cytokines are another possible contributor to the fibrosis of the peritoneal membrane. Chronic inflammation and recurrent peritonitis can induce the migration of inflammatory cells to the site of inflammation. Interleukin 6 (IL-6) released by T cells and macrophages provokes fibrosis [30]. Infiltrating macrophages and lymphocytes release cytokines such as IL-6, interleukin 1β (IL-1β), and tumor necrosis factor-alpha (TNF-α). Together, these cytokines promote fibroblast proliferation and collagen synthesis and eventually lead to peritoneal fibrosis.

For that reason, certain genes that are associated with cytokine activation and interleukins were observed to be expressed higher among patients with long-term PD [30]. Among these genes, lymphocyte antigen 9, chemokine C-C receptor 7 (CCR7), and interleukin 2 receptor alpha (IL-2RA) chain are observed to be expressed higher among patients with long-term PD [30].

Interleukin 17 (IL-17) is another cytokine not only released predominantly by T helper 17 (Th17) lymphocytes but also released by other inflammatory cells such as mast cells [31]. IL-17 can further promote the peritoneal expression of IL-6 [32]. The presence of IL-17 has been investigated extensively in other organs such as in pulmonary inflammation and fibrosis [33]. In peritoneal biopsies obtained from the sub-mesothelium of PD patients, immunostaining has confirmed the presence of IL-17-releasing inflammatory cells [32]. Although recent studies have confirmed the effect IL-17 blockade on the reversal of peritoneal fibrosis, further studies are still required to provide enough evidence on the response of the peritoneum to anti-inflammatory therapeutics and perhaps IL-17 inhibitors.

To prevent, slow down, or possibly reverse peritoneal fibrosis, peritoneal rest has been recommended. Peritoneal rest has been shown to reverse fibrosis. During the perineal rest, the patient often switches to hemodialysis. Other methods such as the avoidance of the use of an acidic PD solution are recommended. In a study, it’s been confirmed that the progression of vascular lesions in peritoneal tissue was suppressed by using a neutral PD solution.

Finally, toll-like receptors (TLRs) and their role in fibrosis were not discussed in this article. Expressed by many cell types, TLRs are another important component of peritoneal fibrosis. Found on mesothelial cells, TLRs can release proinflammatory and profibrotic mediators such as TGF-β, Interleukins, and other cytokines. The reason for not including TLRs in this article is due to the association of TLRs in clearing infections. Although the therapeutic potential of a TLR in preventing PD-related fibrosis [34] was discussed by multiple articles, this will be a separate discussion concerning post-peritonitis fibrosis.

## Conclusions

In conclusion, PF is a complex process, and many factors contribute to the formation of fibrosis. PD solutions with high glucose content, chronic inflammation, inflammatory cytokines, angiogenesis, and mesothelial to mesenchymal transition are factors contributing to the fibrosis of the peritoneum. These factors, as well as stress-induced fibrosis, were discussed in this article.

Research in the last decade or two has provided great insight into the pathophysiology of peritoneal fibrosis. With the continuous increase in patients with ESRD, further research is still required to improve the clinical outcomes and prevent complications that may arise as a result of CAPD. Most articles published on this topic are studies on animal models, and the safety and efficacy of most therapeutics are yet to be determined in the human population. One major limitation is the limited number of individuals receiving PD in the United States, which is reported to be about 10% in 2017.

Although most of the experimental models are promising in preventing or delaying PD-related fibrosis, most of these recommended treatment options require further research. The lack of sufficient data from real PD patients and many inconclusive data make clinicians depend on conservative treatment such as PD holidays, discontinuation of PD, and nutritional support.

Many pathways and triggers are involved in fibrosis. Among these, TGF-β1 is among the most important ones. The inhibition of TGF-β1 among patients receiving PD and disrupting the SMAD signaling pathway have shown promising results in animal models. Further research is still required to show the efficacy of these agents on patients receiving PD.

The results from this finding suggest the involvement of the TGF-β1-VEGF-A pathway as a key role in fibrosis-associated peritoneal neoangiogenesis. These findings are crucial when considering the management strategies for PD patients. The collection of peritoneal biopsy samples remains a major limitation due to the required direct access to the peritoneum. Clinicians are limited to special circumstances such as exploratory laparotomy or when placing or removing the peritoneal catheter to be able to obtain biopsies.

Newer PD solutions have been shown to improve peritoneal viability and reduce the complication that may arise as a result of continuous PD. The effects of either neutral pH, low GDP solution, or icodextrin on patient survival remain inconclusive; further research is required to remove any uncertainty.

One last topic worth mentioning is that with the continuing rise in the incident and prevalence of patients with ESRD, one recent topic that may have an impact on the management of individuals receiving PD is the application of artificial intelligence (AI) or machine learning (ML) algorithms. AI has been applied to the various fields of medicine, and perhaps its application particularly for those receiving PD can better predict the outcome and increase the level of patient’s safety.
